# Epidemiology, Management, and Outcomes of Acute Diverticulitis in King Abdul-Aziz University Hospital, Jeddah, Saudi Arabia

**DOI:** 10.7759/cureus.32615

**Published:** 2022-12-16

**Authors:** Hanan M Bamanie, Nadim Malibary, Nada A Algarni, Jumana O Badawi, Lujain M AlNasser, Khadijah A Almalki, Renad F Alnemari

**Affiliations:** 1 Surgery, King Abdulaziz University, Jeddah, SAU; 2 Visceral and General Surgery, Hautepierre Hospital, Strasbourg, FRA

**Keywords:** outcomes, prevalence, complications, epidemiology, saudi arabia, acute diverticulitis

## Abstract

Background: Acute diverticulitis is considered one of the most common emergencies presenting with acute abdomen. There is a paucity of literature on the epidemiology and clinical picture of acute diverticulitis among the Middle Eastern population. Thus, this study aimed to describe the epidemiology, complications, and outcomes in addition to the management of acute diverticulitis in King Abdul-Aziz University Hospital (KAUH), Jeddah, Saudi Arabia.

Methods: This retrospective study was conducted from 2009 to 2019, using data extracted from an electronic medical system. Data obtained included demographics, clinical presentation, and patient management. Quantitative variables were described as mean and standard deviation, whereas qualitative variables were described as numbers and percentages. The Mann-Whitney U test was used for non-parametric variables, and correlation analysis was done using Spearman’s test.

Results: Forty-five patients with a median age of 53 years had acute diverticulitis. Twenty-eight patients (62.2%) were Saudi Arabians, and 27 (60%) were male. The majority of patients (n=32, 71.1%) had only left-sided disease, and abdominal pain was the most frequently reported symptom (n=35, 77.8%). Computed tomography revealed that 21 (72.4 %) patients had Hinchey classification stage IA disease. The recurrence rate was 24.4% (N =11). Four patients required 30-day readmission (8.9%). The most commonly used inpatient antibiotic was metronidazole, and the most common surgical procedure was Hartmann’s procedure. The 30-day mortality rate was 6.7% (n=3).

Conclusion: This study found that acute diverticulitis is more prevalent in men, has a high recurrence rate, and is predominantly seen in the left colon. Most patients have an uncomplicated form of the disease. Given the lack of previous studies in Saudi Arabia, future research should include population-based studies to identify the prevalence, complications, and outcomes of acute diverticulitis in the country.

## Introduction

Several studies on the diverticular disease of the colon have been reported, however, our understanding of the clinical course of the disorder and its prognosis is limited [1]. Diverticulosis is often asymptomatic and discovered accidentally, it is difficult to accurately estimate its actual prevalence. Nevertheless, the number of cases continues to rise in developed countries in which nearly two-thirds of adults (>18 years of age) develop diverticulosis [2,3]. According to a retrospective study, 7.4% appeared to be the prevalence of colonic diverticulosis in Saudi Arabia, with a mean age of 60 years [3].

Colonic diverticular disease can range from being symptomatic with uncomplicated diverticular disease to being symptomatic with complications. These complications include acute diverticulitis, which is inflammation triggered by the obstruction of the diverticular ostium owing to stool fragments or food particles [4,5]. Acute diverticulitis is one of the most encountered bowel emergencies that present with acute abdominal pain, accounting for 3.8% of all patients who present to the emergency department with abdominal pain [5].

Other complications include diverticular perforation, which leads to peritonitis, and diverticular hemorrhage, which occurs due to the rupture of diverticula-associated arteries, thereby causing colonic bleeding [4]. In addition, abscesses develop when pus is formed inside the diverticula [4].

Based on our review of studies on diverticular diseases in the Middle East, there are limited data on the epidemiology, management, and complications of acute diverticulitis in Saudi Arabia [3,6]. Therefore, this study aims to describe the epidemiology, complications, and outcomes in addition to the management of acute diverticulitis in King Abdul-Aziz University Hospital (KAUH), Jeddah, Saudi Arabia.

## Materials and methods

This retrospective study was carried out at KAUH, a tertiary center in Jeddah, Saudi Arabia, from 2009 to 2019. The study was approved by the Research Ethics Committee of KAUH (reference No. 473-20). Adults older than 18 years, who were diagnosed with a proven diverticular disease through computed tomography (CT) or colonoscopy and who were admitted and treated in KAUH, were included; all the data used were extracted from the electronic medical system. We identified 45 patients with acute diverticulitis, who were included in the study. The data obtained included demographics and clinical data regarding comorbidities, risk factors, age at diagnosis, presenting symptoms, number of acute attacks, colonoscopy results, length of hospital stay (LOS), 30-day readmission, hospital mortality, and treatment method (medical or surgical). CT findings were described using the Hinchey classification system. Microsoft Excel, version 16.59, was used for data entry, and Statistical Package for the Social Sciences (SPSS), version 26, was used for statistical analysis. Numbers and percentages were used to represent qualitative variables. Quantitative variables were described as mean and standard deviation (mean SD), and non-parametric variables were tested using the Mann-Whitney (U) test. Spearman’s test was used for correlation analysis, and a p-value of 0.05 was considered statistically significant.

## Results

The median age of the 45 patients was 53 years. Of all the patients, 28 (62.2%) were Saudi Arabians and 17 (37.8%) were non-Saudi Arabian, 27 (60%) were males, 1 (2.2%) was an alcohol consumer, and 4 (8.9%) were smokers. The most common comorbidities were hypertension (n=17, 37.8%), diabetes mellitus (n=13, 28.9%), and ischemic heart disease (n=7, 15.6%). The mean white blood cell (WBC) count was 10.39 ± 5.71 K/µL (reference range 4.5-11.5), C-reactive protein (CRP) level was 47.96 ± 74 mg/L (reference range 0-3), and erythrocyte sedimentation rate (ESR) was 15 ± 0.001 mm/H (reference range 1-20) (Table 1).

**Table 1:** Distribution of the studied patients according to their characteristics CRP: C-reactive protein; ESR: Erythrocyte sedimentation rate

Variable	No. (%)
Age	53
Sex	
Female	18 (40)
Male	27 (60)
Nationality	
Non-Saudi Arabian	17 (37.8)
Saudi Arabian	28 (62.2)
Alcohol consumption	
No	6 (13.3)
Unknown	38 (84.4)
Yes	1 (2.2)
Smoking	
No	17 (37.8)
Unknown	24 (53.3)
Yes	4 (8.9)
Comorbidity	
Diabetes mellitus	13 (28.9)
Hypertension	17 (37.8)
Ischemic heart disease	7 (15.6)
Chronic kidney disease	4 (8.9)
Hyperlipidemia	4 (8.9)
Anemia	2 (4.4)
Laboratory results	
White blood cell count	10.39 ± 5.71 K/uL
CRP	47.96 ± 74 mg/L
ESR	15 ± 0.001 mm/H

**Figure 1:**
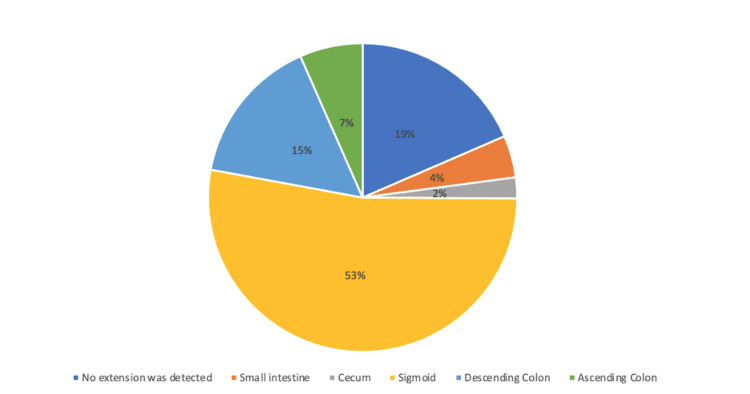
Percentage distribution of studied patients according to extension of diverticular disease

Regarding the disease site, 32 (71.1%) patients had only left-sided disease, whereas in 52% of the patients, the disease was in the sigmoid colon (Figure 1). The most reported symptoms among the patients were abdominal pain (n=35, 77.8%), nausea (n=12, 26.7%), vomiting (n=11, 24.45%), and fever (n=9, 20%). Moreover, 34 (75.6%) patients had only one diverticulitis attack, and 11 (24.4%) had more than one attack. Four patients required 30-day readmission (8.9%). One patient had an abscess and perforation, one had an abscess and a colovesical fistula, one had bleeding, and one was readmitted for the failure of medical treatment. The 30-day mortality rate was 6.7% (n=3), and the intensive care unit admission rate was 11.1% (n=5). More than half of the patients were followed up in the clinic and treated as an outpatient (n=23, 51.1 %) (Table 2).

**Table 2:** Distribution of the studied patients according to their clinical data CT: computed tomography

Variable	No. (%)
Presenting symptoms	
Abdominal pain	35 (77.8)
Nausea	12 (26.7)
Vomiting	11 (24.4)
Fever	9 (20)
Lower gastrointestinal bleeding	8 (17.8)
Diarrhea	6 (13.3)
Not found	6 (13.3)
Chronic constipation	5 (11.1)
Melena	4 (8.9)
Loss of appetite	3 (6.7)
Abdominal distension	2 (4.4)
Weight loss	2 (4.4)
Number of diverticulitis attacks	
1 attack	34 (75.6)
2 to 3 attacks	9 (20)
More than 3	2 (4.4)
30-day readmission (due to complications)	
No	41 (91.1)
Yes	4 (8.9)
Cause of 30-day readmission	
Abscess and perforation	1 (25)
Abscess and colovesical fistula	1 (25)
Bleeding	1 (25)
Failure of medical treatment	1 (25)
Hospital mortality (30-day mortality)	
No	42 (93.3)
Yes	3 (6.7)
Subsequent admissions	
Second admission	8 (17.7)
Third admission	3 (6.6)
Follow-up (outpatient clinic)	
No	22 (48.9)
Yes	23 (51.1)
Intensive care unit admission	
No	41 (91.1)
Yes	5 (11.1)
CT scan performed	
No	16 (35.6)
Yes	29 (64.4)
CT, Hinchey classification	
Stage Ia: phlegmon	21 (72.4)
Stage Ib: diverticulitis with pericolic or mesenteric abscess	7 (24.1)
Stage II: diverticulitis with walled off pelvic abscess	1 (3.5)
Was colonoscopy performed before the diagnosis?	
No	38 (84.4)
Yes	7 (15.6)
What were the colonoscopy findings (performed before diagnosis)?	
Erythema adjacent	1 (14.2)
Luminal narrowing	1 (14.2)
Diverticula	2 (28.8)
Angiodysplasia	1 (14.2)
Not found	2 (28.8)
Was colonoscopy performed after the diagnosis?	
No	24 (53.3)
Yes	21 (46.7)
Disease location	
Both right and left	1 (2.2)
Left	32 (71.1)
Not found	7 (15.6)
Right	3 (6.7)
Small intestine	2 (4.4)

**Figure 2:**
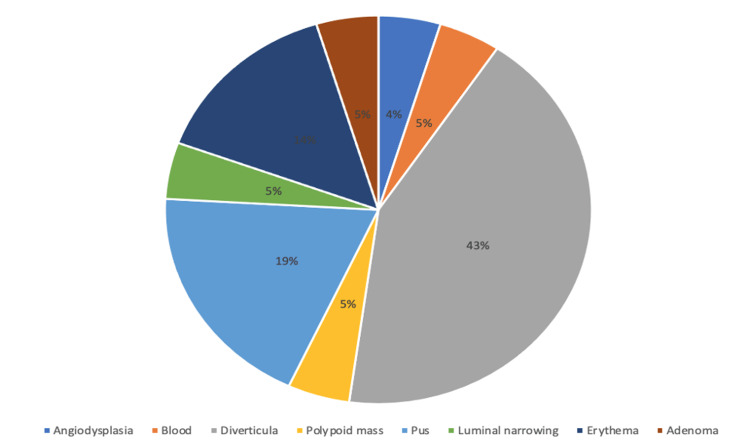
Colonoscopy findings (performed after the diagnosis)

Regarding the Hinchey classification of the CT findings, most of the patients (n=21, 72.4%) were classified as having stage Ia disease (phlegmon). Furthermore, 21 patients (46.7%) underwent colonoscopy after the diagnosis. The most common findings were diverticula (42.8%), pus formation (19%), and erythema (14.7%) (Figure 2). Surgery was performed in 10 patients (22.2%), of whom six (60.0%) underwent emergent surgery (immediate action) and four (40.0%), urgent surgery (within 24 hours). The most common surgical procedure was Hartmann’s procedure (n=6, 54.5%). Local perforation with abscess formation was the most common indication for surgery (n=5; 45.5%). Only one patient had a wound infection, and one had a urinary tract infection after surgery (Table 3).

**Table 3:** Distribution of the studied patients according to their management data (n=45)

Variable	No. (%)
What were the treatment methods?	
Antibiotics	32 (71.1)
Antibiotics + Percutaneous drainage	3 (6.7)
Surgery	10 (22.2)
Was surgery performed?	
No	35 (77.8)
Yes	10 (22.2)
If antibiotics were necessary, what type of antibiotic was administered?	
Ceftriaxone	10 (22.2)
Metronidazole	16 (35.6)
Piperacillin + Tazobactam	11 (24.4)
Meropenem	3 (6.7)
Co-amoxiclav	1 (2.2)
Imipenem	3 (6.7)
Cefuroxime	7 (15.6)
Ciprofloxacin	3 (6.7)
Cyclosporine	1 (2.2)
If antibiotics were necessary, what was the discharged antibiotic administered?	
Metronidazole and Cefuroxime	9 (41%)
Metronidazole and Ciprofloxacin	5 (22.7%)
Ciprofloxacillin	2 (9%)
Co-amoxiclav	2 (9%)
Ciprofloxacillin and Co-amoxiclav	1(4.5%)
Ciprofloxacillin and Clindamycin	1 (4.5%)
Metronidazole	1(4.5%)
Ciprofloxacillin, Metronidazole, and Co-amoxiclav	1 (4.5%)
Antibiotics duration (mean + standard deviation) For how many days were hospital antibiotics administered?	8.1 ± 5.43
Ciprofloxacin duration	11.9 ± 9.9
Metronidazole duration	10.06 ± 7.82
Cefuroxime duration	7.62 ± 1.68
Co-amoxiclav duration	11.33 ± 8.38
If surgery was necessary, what was the type of surgery (n=11)?	
Emergent	6 (60.0)
Urgent	4(40.0)
What was the surgical procedure used? (n=11)	
Colectomy with primary anastomosis with covering loop ileostomy	2 (18.1)
Exploratory laparotomy	2 (18.1)
Hartmann's procedure	6 (54.5)
Laparoscopic left hemicolectomy	1 (9.1)
What was the cause/indication of surgery?	
Perforation with general peritonitis	1 (9.1)
Local perforation and abscess	5 (45.5)
Abscess	1 (9.1)
Large bowel obstruction	1 (9.1)
Recurrent diverticulitis	2 (18.1)
Bladder fistula	1 (9.1)
Were there any complications after surgery?	
Wound infection	1 (10)
Urinary tract infection	1 (10)
Length of hospital stay (median)	4 days

Regarding antibiotics, 32 patients (71.1%) were treated with antibiotics alone, which was empirical. The most common inpatient antibiotics used were as follows: metronidazole (n=16, 35.6%), Tazocin (n=11, 24.4%), and ceftriaxone (n=10, 22.2%). Regarding discharge antibiotics, the most common antibiotics used were as follows: metronidazole and cephalosporin (n=9; 41%; mean duration, 8.4 days); metronidazole and fluoroquinolones (n=5; 22.7%; median duration, nine days); fluoroquinolones alone (n=2; 9%; duration, seven days in both patients), followed by Augmentin (n=2, 9%) in one patient, for 17 days, and in the other for seven days; fluoroquinolones and Augmentin (n=1; 4.5%; duration, 21 days); fluoroquinolones and clindamycin (n=1; 4.5%; duration, seven days); metronidazole alone (n=1; 4.5%; duration, three days); and fluoroquinolones, metronidazole, and Augmentin (n=1; 4.5%; duration, six days) (Table 3). The median LOS was four days.

## Discussion

This retrospective study conducted in Saudi Arabia showed that the mean age of patients with acute diverticulitis was 55 years, which is higher than that of patients in Kuwait and South Korea and lower than that of patients in Germany [3,7,8]. Several studies have reported that the location of diverticular diseases is different between Eastern and Western countries. The data show that the disease is predominantly seen in the left colon in Western populations, whereas the right colon is reported to be more frequently affected in Asian populations [9-11]. The majority of our patients had the left-sided disease, with the sigmoid colon affected in more than half of them; this finding is similar to that in Western countries. This may be related to the fact that food habits in Saudi Arabia are similar to those in Western countries, with high consumption of red meat and a low-fiber diet [3]. According to our study, abdominal pain was the most frequent presenting symptom, which was seen in 77.8% of the patients. Similarly, in the United States, the most predominant presentation on emergency admission was localized abdominal pain in 80% of the patients [12]. Moreover, in Italy, the most reported symptoms were localized abdominal pain and fever which were seen in 47.88% of the patients [13].

The median LOS in our study was four days, which is nearly equivalent to the mean LOS in Kuwait and the United States (5.3 ± 4.5 and 5.3 ± 12.0, respectively) [6,14]. In our study, the recurrence rate was 24.4%. In Kuwait, the recurrence rate is 18.9% [6]. In comparing the recurrence rate between the Western and Middle Eastern populations, the recurrence rate was found to be lower in the Middle East, with one recurrence occurring in 9% of cases and more than one occurring in 3% [15]. The proportion of patients who were readmitted within 30 days was 8.9%, which is comparable to that in a cohort study, reporting that 8.6% of patients required an unplanned 30-day readmission [16].

It is estimated that up to 25% of patients with acute diverticulitis are associated with either acute or chronic complications, among which diverticular abscesses are the most common and occur in approximately 17% of the cases [17]. These findings are similar to our findings, which showed that nine patients (20%) had complications, with abscesses and local perforation being the most common (55%). The estimated proportion of patients who underwent surgery at the time of admission was 25% [18]. Similarly, in our study, 22% of patients underwent surgery at the time of admission. The limitations of this study include the small sample size as it was carried out at a single tertiary center in the country.

## Conclusions

In our study on 45 patients with acute diverticulitis, the mean age was 55, the disease was predominantly seen in the left colon and had a high recurrence rate of 24% and a 30-day readmission rate of 8.9%. Surgical intervention was performed in 10 patients, and complications were reported in only two patients, with one having a wound infection, and the other with a urinary tract infection. Most patients had an uncomplicated form of the disease, with antibiotics being the most common method of treatment. Given the lack of previous studies in Saudi Arabia, future research should involve population-based studies for the identification of the prevalence, complications, and outcomes of acute diverticulitis in the country.
